# Eight recommendations for advancing blue Nature-based Solutions

**DOI:** 10.1007/s13280-026-02380-4

**Published:** 2026-04-04

**Authors:** Bethan C. O’Leary, Catarina Fonseca, Gema Casal, Cindy C. Cornet, Fabiola Espinoza Córdova, Elisa Furlan, Julie P. Hawkins, Silvia de Juan, Torsten Krause, Géraldine Pérez, Rémy Simide, Ewan Trégarot, Callum M. Roberts

**Affiliations:** 1 Centre for Ecology and Conservation, Faculty of Environment, Science and Economy, University of Exeter, Penryn Campus, Penryn, Cornwall, TR10 9FE UK; 2 cE3c – Centre for Ecology, Evolution and Environmental Changes and CHANGE – Global Change and Sustainability Institute, Faculdade de Ciências, Universidade de Lisboa, Portugal, Lisbon, Portugal; 3 MARE – Marine and Environmental Sciences Centre / ARNET — Aquatic Research Network, Faculdade de Ciências, Universidade de Lisboa, Portugal, Lisbon, Portugal; 4 A Coruña Oceanographic Centre (IEO-CSIC), Pº Marítimo Alcalde Francisco Vázquez, 10, 15001 A Coruña, Spain; 5 National Centre for Geocomputation, Maynooth University, Co. Kildare, Maynooth, Ireland; 6 Centre for Blue Governance, University of Portsmouth, Richmond Building, Portland Street, Portsmouth, PO1 3DE UK; 7 Lund University Centre for Sustainability Studies, P.O. Box 170, 221-00 Lund, Sweden; 8 CMCC Foundation - Euro-Mediterranean Center on Climate Change, Via Della Libertà 12, 30175 Venice, Italy; 9 Department of Environmental Sciences, Informatics and Statistics, Venice Ca’ Foscari University, Via Torino 155, 30172 Venice, Italy; 10 The Mediterranean Institute for Advanced Studies (IMEDEA-UIB-CSIC), Miquel Marques 21, 07190 Balearic Islands, Spain; 11 Oceanographic Institute Paul Ricard, Île des Embiez, 83140 Six Fours les Plages, France

**Keywords:** Marine and coastal social-ecological systems, Nature’s contribution to People, NbS, Societal challenges, Sustainable development, Transformative change

## Abstract

Crises narratives surrounding the societal challenges of biodiversity loss, climate change, and human health and well-being dominate current debates in global environmental politics. These challenges are deeply interconnected and require transformative and integrated change. Marine and coastal Nature-based Solutions (blue NbS) are an opportunity to minimise harmful human impacts on these ecosystems while providing co-benefits to people and nature. However, uptake of the approach is currently slow despite the urgency of addressing global challenges. For blue NbS to become a key tool for environmental management, integrated social-ecological advice on action for practitioners is required, alongside guidance for new research. Here, we present eight recommendations to help advance further research, development, and implementation of effective blue NbS. Recommendations are broadly grouped into three themes: (1) objectives, (2) function, and (3) mechanisms. Through these, we aim to encourage dialogue into strategic blue NbS implementation and research that supports solution-focussed narratives.

## Introduction

Narratives of crisis surrounding the societal challenges of biodiversity loss, climate change, and human health and well-being dominate current discourses in environmental politics (Pörtner et al. 2023). Rapidly decarbonising societies and reducing greenhouse gas emissions are critical for climate action (IPCC 2022). However, climate change and other societal challenges are deeply interconnected and cannot be tackled without transformative and integrative approaches to protect and restore nature (IPCC 2022; IPBES 2022).

Nature-based Solutions (NbS) aim to simultaneously address biodiversity loss alongside other societal challenges by integrating protection, restoration, and sustainable management measures to holistically manage human impacts that can degrade ecosystems and, consequently, maximise nature’s contributions to people (IUCN 2020; O’Leary et al. 2023). Building on ecosystem-based approaches to environmental management, marine and coastal (blue) NbS can support practitioners to design interventions suited to complex social-ecological systems, where ecological processes, governance arrangements, and social values interact (O’Leary et al. 2023; Fonseca et al. 2023). In such contexts, blue NbS emphasise participatory and inclusive approaches. However, communities and stakeholder groups cannot be assumed homogeneous, instead internal diversity and power relations need recognition and participatory approaches require attention to representation, inclusion, and potential risks such as marginalisation.

**Fig. 1:**
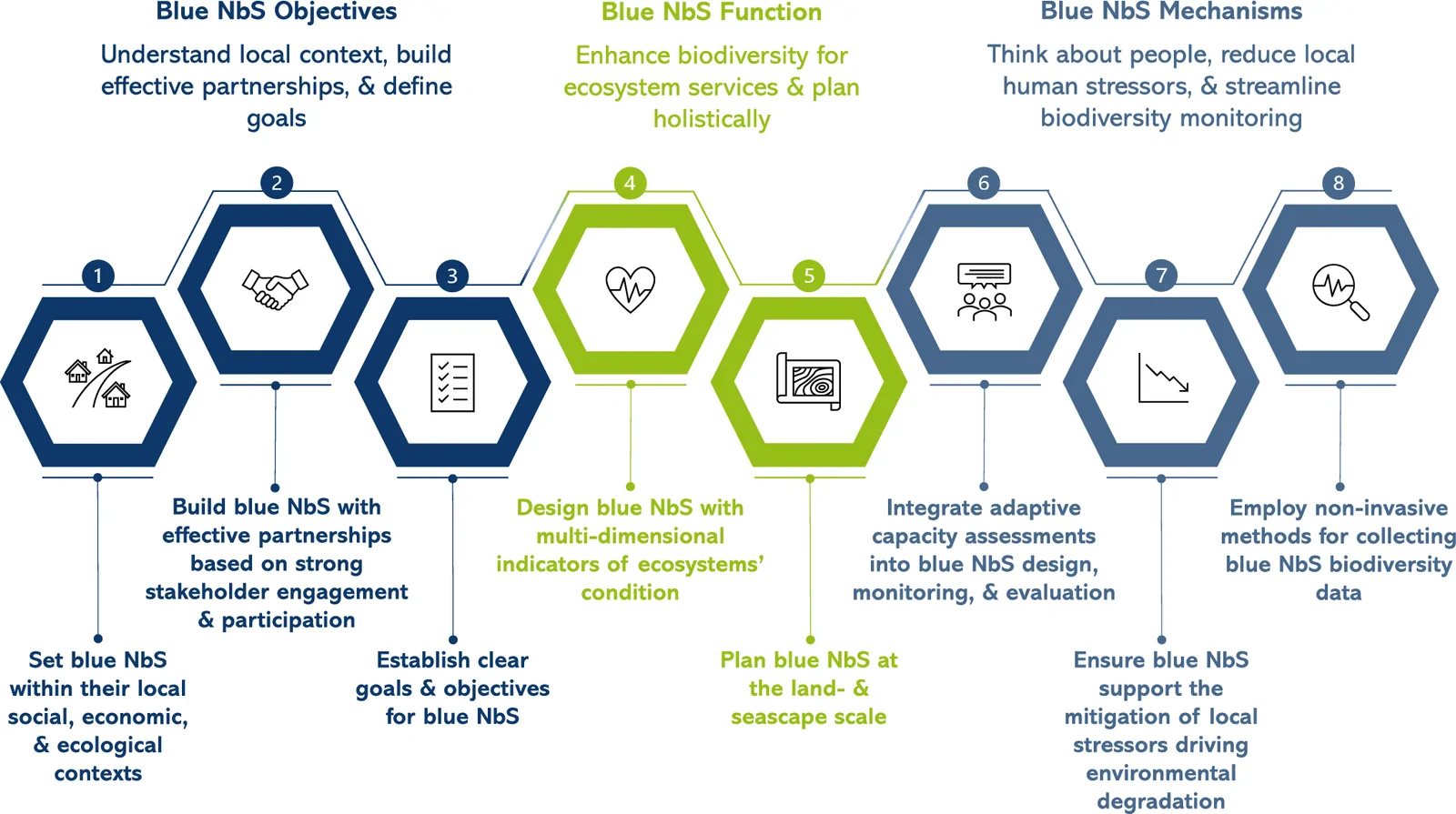
Eight recommendations to help advance blue Nature-based Solutions (NbS)

Despite their potential, blue NbS are underused, with most NbS in terrestrial and urban settings (O’Leary et al. 2023). Nonetheless, existing ecosystem-based approaches can provide insights for improving blue NbS effectiveness. Here, we present eight recommendations to advance blue NbS, informed by research from the European Horizon 2020 project ‘MaCoBioS’^1^ and developed through reflection on the project’s empirical case studies, stakeholder workshops, and relevant literature, rather than formal systematic review or case selection. These recommendations are broadly grouped into the themes of (1) objectives, (2) function, and (3) mechanisms, which together provide guidelines for practitioners and researchers on crucial areas of blue NbS design and implementation (Fig. 1).

The recommendations draw on social-ecological, resilience, and co-management thinking, framing blue NbS as adaptive, multi-actor interventions within coupled human–environment systems. Terms such as ecosystem health, effectiveness, and co-benefits are used as context-dependent, practice-oriented concepts and interpreted through a social-ecological lens, rather than as fixed or universally agreed metrics. Accordingly, recommendations emphasise inclusivity, sustainability, and environmental awareness, offering a new perspective on responsibly integrating blue NbS into environmental management and supporting solution-focussed action for more resilient ecosystems and societies in the short- and long-term. Our recommendations are summarised in the following sections.

## Theme 1: Focus on objectives—Understand local context, build effective partnerships, and define goals

### Recommendation 1: Set blue NbS within their local social, economic, and ecological contexts

Blue NbS advance existing ecosystem-based management approaches by taking a cohesive social-ecological approach, embedding transparency, participation, and equity into decision-making from the outset (IUCN 2020; O’Leary et al. 2023). Blue NbS bridge societal goals, helping identify desired outcomes, choose locally appropriate interventions to reach outcomes, and integrate top-down (state-led) and bottom-up (stakeholder-led) approaches to management (Gaymer et al. 2014). Although stakeholder-led efforts may take time to influence policy, combining them with state-led actions can be successful. For example, while it took thirteen years of local community advocacy before Scotland’s first marine no-take zone was legally established, this protected area is now internationally recognised for its conservation success (Stewart et al. 2020). To scale up action, stronger capacity building, collaboration between stakeholders and policymakers, and incentives are required for sustained, meaningful, inclusive, and tailored participation (Sterling et al. 2017), recognising that such conditions may be unevenly available across contexts (see also Recommendations 2, 3, and 6).

Blue NbS need to adopt a pluralistic approach to foresee, prevent, and manage local environmental impacts (Welden et al. 2021). This means integrating different intervention types, management approaches, and opinions as to which NbS are most locally appropriate. Strategies for blue NbS management may include layering multiple interventions to facilitate short- and long-term benefits to ecosystems, local communities, and other stakeholders. For example, combining sustainable seaweed farming with a marine protected area in Zanzibar has supported ecosystem protection and local livelihoods (Tallent and Zabala 2024). The most appropriate intervention depends on an ecosystem’s condition and biophysical properties, as well as surrounding human activities and impacts, and societal acceptability and feasibility (Pérez et al. 2024). Active restoration, for instance, can be effective in particular conditions for fast-growing species, depending on the level of degradation and exposure to pressures (Saunders et al. 2020). However, most ecosystems benefit from passive restoration measures that reduce the source of degradation (Saunders et al. 2020). Certainly, framing interventions around ecological boundaries and human–environment interactions through blue NbS should help align stakeholder values, deliver ecosystem and societal benefits, facilitate compromises, encourage participation, and respond to calls for greater reflexivity in conservation science (Saunders et al. 2020; Montana et al. 2020; Tallent and Zabala 2024).

Existing management processes, vested interests, and cultural factors may pose both challenges and opportunities for blue NbS, with contextual dynamics shaping implementation strategies and their effectiveness (Sterling et al. 2017). Local contexts and knowledge shape how a particular land- and seascape is used, managed, and valued (Sterling et al. 2017; Pascual et al. 2023). Understanding context is crucial for informing effective, equitable approaches, and stakeholders play a vital role, contributing: insights on ecosystem conditions; impacts arising from human activities on people and ecosystems; management challenges; and opportunities to improve social-ecological health. Management measures must align with ecological boundaries and be driven by the people whose actions ultimately shape success or failure. This includes the support of governing institutions (e.g. Gill et al. 2017) alongside local stakeholder engagement and co-management (Sterling et al. 2017) to yield positive social and conservation outcomes.

### Recommendation 2: Build blue NbS with effective partnerships based on strong stakeholder engagement and participation

Experience shows that intervention success hinges on local community and stakeholder support, along with enabling conditions like supporting legislation, explicit objectives, regulatory enforcement and compliance, adequate resources, and appropriate spatial scale (Gill et al. 2017; Saunders et al. 2020; Pascual et al. 2023). Early and meaningful stakeholder engagement is key (Moraes et al. 2022; Tallent and Zabala 2024), with trust hard to regain following stakeholder exclusion (Cvitanovic et al. 2021). For blue NbS to deliver a step-change in marine and coastal social-ecological management, broad stakeholder engagement, cross-sectoral collaboration, and robust public–private–community partnerships are essential, though often challenging to sustain given time, capacity, and political constraints. Indeed, practitioners and researchers alike emphasise that insufficient integration of social sciences and ineffective stakeholder engagement are major barriers to blue NbS implementation (O’Leary et al. 2023, 2024).

Stakeholder mapping helps identify relevant and diverse stakeholders, their influence on decision-making, and modes of engagement. Numerous global examples highlight the importance of including diverse stakeholders when developing ecosystem-based management actions (Gaymer et al. 2014). For example, native Hawaiians were key to the Papahānaumokuākea Marine National Monument being designated partly for reasons of cultural heritage (Kikiloi et al. 2017), while local community pressure led to Scotland’s first no-take zone (Stewart et al. 2020). Integrating stakeholders effectively and overcoming power dynamics can transform poorly managed areas into best-practice case studies, as in the Italian Torre Guaceto marine protected area (Russi 2020). Diverse representation will help blue NbS incorporate fresh perspectives and innovative approaches, while integrating livelihood considerations with conservation needs can help ensure stakeholders feel their values and priorities are respected. It will also empower stakeholders to shape blue NbS policies, jointly set objectives, and build wider support for management (Stewart et al. 2020; Tallent and Zabala 2024) (Recommendations 1, 3, 6). Effective participation of diverse stakeholders is a long-term investment that can ultimately lower costs and improve the cost-effectiveness of interventions (Ponce Reyes et al. 2019). Note though, estimating cost-effectiveness is complex due to differing actor perceptions, timescales, and (in)direct, (in)tangible, and unpredictable outcomes (e.g. Anggraeni et al. 2019). Simply increasing stakeholder engagement without considering the participation process or its value will, therefore, not suffice for blue NbS to deliver improvements on existing practices.

Genuine collaboration between researchers, societal groups, and policymakers is challenging, requiring cultural integration, shared interests, relationship-building, and overcoming time, capacity, and language barriers (Schoonover et al. 2019). Therefore, engagement methods (e.g. workshops, advisory bodies, etc.) should differ depending on whether sharing information, consulting, or promoting active participation and co-creation. To prevent stakeholder fatigue (Sterling et al. 2017), it is crucial to acknowledge efforts, manage expectations, ensure accessibility, and make participation meaningful. In cases where high public involvement was followed by unilateral decision-making, stakeholders were left feeling disillusioned and disenfranchised (De Santo 2016; Sterling et al. 2017). Despite these challenges, effective blue NbS engagement pathways remain key for enabling stakeholders to become involved and invested in decision-making (Sterling et al. 2017; Schoonover et al. 2019).

### Recommendation 3: Establish clear goals and objectives for blue NbS

Blue NbS may have diverse ecological, social, and economic objectives, but all must be desirable, at least to some, viable, and feasible, with trade-offs often requiring prioritisation among competing goals. However, they cannot address all societal challenges in all localities. The goals (i.e. broad long-term outcomes) and objectives (i.e. shorter-term measurable actions to achieve a goal) set by practitioners and stakeholders for specific blue NbS will shape their design, monitoring, and evaluation (Gómez Martín et al. 2020; EC: DG RTD 2021). For a blue NbS to be effective, it must achieve its intended objectives; to be cost-effective, outcomes must justify the economic costs (EC: DG RTD 2021). To allow an understanding of when blue NbS succeed or fail and why, clear, unambiguous objectives related to management goals are required (EC: DG RTD 2021). For example, a management goal might be the sustainable exploitation of a fish species, with the objective being to increase its population biomass above sustainable levels. Management goals and objectives should emerge from stakeholder-led problem identification, informing whether a blue NbS is appropriate and, if it is, how to design the intervention and where to place it (Pérez et al. 2024) (Recommendations 1, 2, 5, 6, and 7). In turn, clear objectives will also inform the baseline assessments required before implementing any action. Poorly defined goals and objectives have previously prevented action prioritisation, evaluation of environmental plans, and organisational learning (e.g. Domínguez-Tejo and Metternicht 2018).

**Fig. 2:**
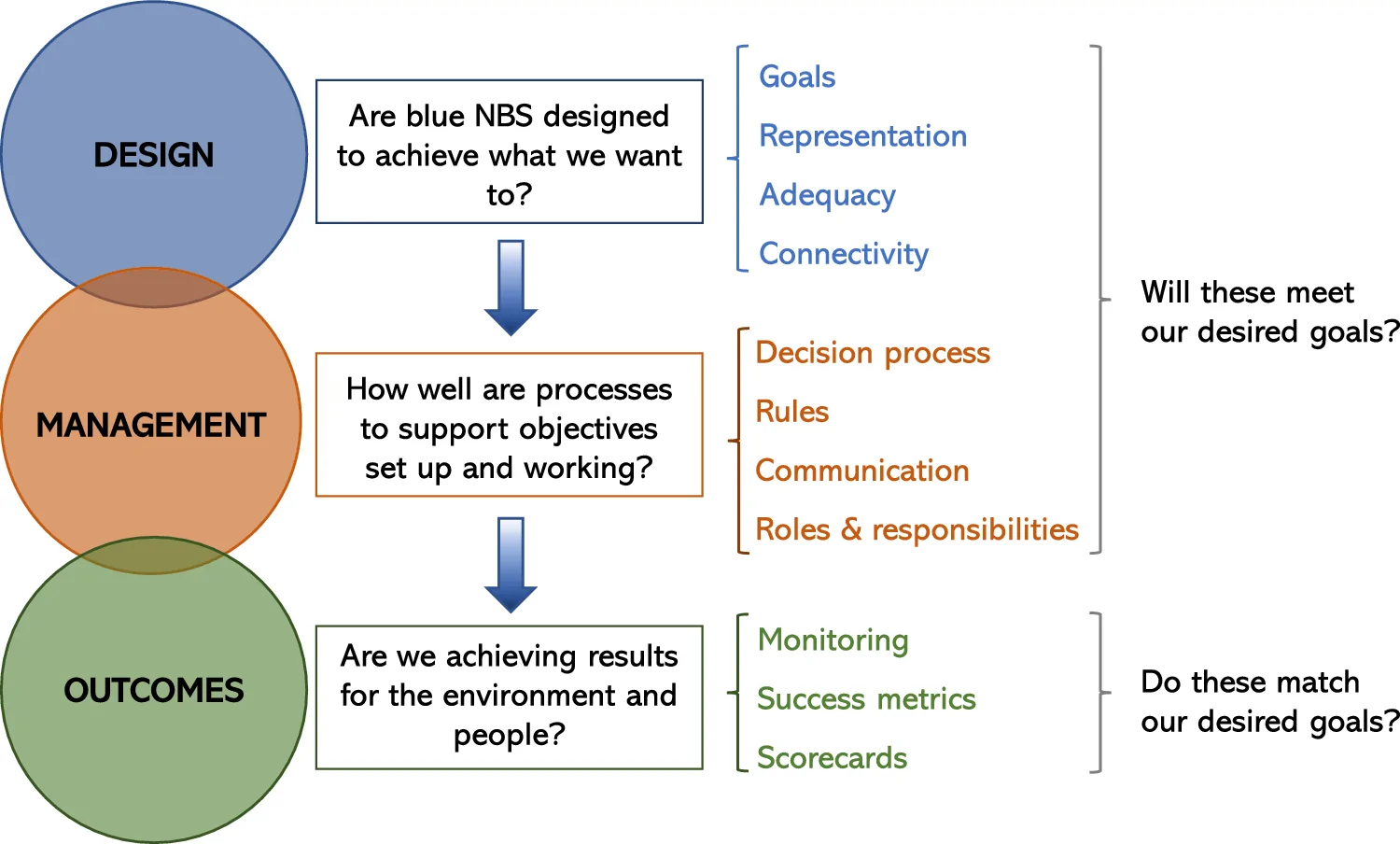
Factors to consider when evaluating intervention effectiveness

Various factors influence intervention effectiveness, including its design (e.g. size, location, species/habitats representation) and management (e.g. decision processes, capacity), with effectiveness understood here as the extent to which stated social-ecological objectives are achieved, rather than as a single standardised outcome (Fig. 1). Moreover, given that interventions can result in trade-offs, benefits, or disbenefits (Gómez Martín et al. 2020), effectiveness evaluations must consider outcomes (e.g. intervention impacts) against social-ecological objectives, including for different stakeholder groups. This makes it even more important that the objectives informing evaluation are stakeholder-led and based on genuine collaboration and diverse participation (Recommendation 2). To support adaptive management, blue NbS require a long-term strategy for monitoring and evaluating the effectiveness of an intervention’s design, management, and ecological, social, economic, and institutional outcomes (Fig. 2). While we know less about how to measure and attribute causality for social, economic, and institutional outcomes than environmental ones (EC: DG RTD 2021; O’Leary et al. 2021), planning blue NbS with clear objectives coupled with careful consideration of appropriate indicators and monitoring and evaluation strategies (Recommendations 4 and 6) will help address this.

## Theme 2: Recognise blue NbS function—Enhance biodiversity for ecosystem services and plan holistically

### Recommendation 4: Design blue NbS with multi-dimensional indicators of ecosystems’ conditions

Marine and coastal ecosystems in good ecological condition (often referred to as ‘ecosystem health’, a multi-dimensional and context-dependent concept encompassing ecosystem extent, connectivity, structure, and biodiversity) are critical for ensuring continued ecosystem services provision (IPBES 2022). Blue NbS protect and restore ecosystems to improve their health and functionality. Substantial uncertainties exist when it comes to quantifying the relationship between ecosystem health and services provision (O’Leary et al. 2023; Casal and McCarthy 2023), as well as in defining ecosystem health itself (Lu et al. 2015). However, we know that if ecosystems are in good ecological condition, they are more resilient to perturbations and environmental change, irrespective of whether changes originate from local stressors such as direct human activities or from climate change and its associated effects (e.g. Gove et al. 2023; Trégarot et al. 2024) (Recommendation 7). Therefore, for an intervention to qualify as a blue NbS, it must be designed to improve ecosystem health and human well-being by supporting and enhancing biodiversity, ecosystem function, and connectivity while also contributing to alleviate other societal challenges (IUCN 2020).

Assessing the ecological condition of an ecosystem is essential to inform the type of actions to be implemented and their design (Pérez et al. 2024) (Recommendation 8). To understand how to design a blue NbS to support healthy ecosystems, and to assess whether any implemented intervention achieves this, regular and long-term monitoring and evaluation are necessary (Recommendation 3). Assessments of ecological condition require indicators to be interpreted based on context (e.g. ecosystem, species, geomorphic settings) and against a baseline to compare with (i.e. an historical point or reference ecosystem), which can be defined by local knowledge, scientific evidence, and expert judgement (e.g. de Juan et al. 2018).

As of now, no single indicator can assess an ecosystem’s condition as it derives from a composite of factors including physical environment, species diversity, trophic level, and human pressures (Teixeira et al. 2016). Because of this multi-dimensionality, ecosystem condition assessments comprising a suite of complementary indicators are essential for blue NbS design, monitoring, and evaluation. However, integrating these factors for comprehensive assessments is challenging, as demonstrated, for example, by the application of the European Union’s Marine Strategy Framework Directive (Borja et al. 2013). Furthermore, it is important to recognise that the choice of suitable indicators to investigate ecosystem health will be determined by factors that include: the ecosystem(s) of interest; local social-ecological context; data availability; capacity for data collection and analysis; and the intervention’s objectives.

### Recommendation 5: Plan blue NbS at the land- and seascape scale

Blue NbS advance ecosystem-based management by explicitly incorporating social-ecological interactions. In marine and coastal systems, this is especially challenging because high ecological connectivity and varied spatial distributions create a dynamic continuum of interconnected habitats, species, and ecosystems, where impacts from human activities and management in one place can affect people and nature in another (O’Leary and Roberts 2018; Goodridge Gaines et al. 2022; Vozzo et al. 2023). Hence, blue NbS must adopt a dynamic land- and seascape perspective to effectively support biodiversity, ecosystem service provision, and human well-being (Goodridge Gaines et al. 2022; Vozzo et al. 2023) (Recommendations 6 and 8).

Adopting systems thinking throughout a blue NBS project cycle will allow for the development of synergistic and inclusive management strategies, help anticipate and identify potential co-benefits and trade-offs, and foster continual reflection and learning (Gómez Martín et al. 2020). Here, co-benefits and trade-offs are understood as context-specific social, ecological, or economic outcomes that may accrue unevenly across stakeholders. This approach will also help avoid unforeseen consequences from external activities that could hinder blue NbS effectiveness, and allow for mitigating risks blue NbS may present to people and nature beyond their boundaries. For example, species richness in the Mediterranean Menorca Channel is highest at the seascape scale when “specialist” (e.g. maërl) and “generalist” (e.g. bare sand) habitats co-occur (de Juan et al. 2023). Therefore, focussing blue NbS only on species-rich specialist habitats, without considering connected ecosystems and ecological interactions, could disrupt species–habitat interactions and reduce biodiversity despite management efforts. Similarly, recognising that temporal, spatial, social, ecological, and economic trade-offs are common in marine and coastal management will help move towards a land- and seascape planning model that improves procedural transparency and equity (Fortnam et al. 2023).

Land- and seascape planning undoubtedly adds complexity to decision-making (Murphy et al. 2021; O’Leary et al. 2024). To capitalise on the full potential of blue NbS through joined-up management, the roles, remits, and objectives of stakeholders and organisations involved in any action, together with clear budgets and budgetary responsibility, need to be agreed upon. Despite these challenges, numerous examples of integrated land- and seascape management exist (e.g. Ridge-to-Reef initiatives, Biosphere Reserves, World Heritage Sites), demonstrating that multilevel governance, community empowerment, funding diversification, and ecosystem benefits can be achieved (Murphy et al. 2021; Karimova and Lee 2022). They also show that such approaches can facilitate understanding of other stakeholders’ values and roles in achieving shared goals, as seen in the Xinshe Socio-Ecological Production Landscape and Seascape, Taiwan (Karimova and Lee 2022). By broadening traditional planning perspectives and integrating diverse stakeholders transparently and equitably during the design stage, blue NbS will be able to better consider interdependencies between ecosystems and actions and facilitate the integration of smaller-scale, distributed interventions into a strategic plan to optimise short- and long-term outcomes (Recommendations 1–3, and 6).

## Theme 3: Plan management mechanisms—Think about people, reduce local human stressors, and streamline biodiversity monitoring

### Recommendation 6: Integrate social adaptive capacity assessments into blue NbS design, monitoring, and evaluation

Climate change has disproportionate and differentiated effects across societies and communities. Within each specific situation, people’s capacity to adapt to change will be influenced by local contexts (Whitney et al. 2017; Nielsen et al. 2023). Ecosystem-based marine and coastal management has sometimes negatively impacted people’s well-being and support for actions through inequitable decision-making and uneven distribution of costs and benefits (e.g. De Santo 2016; Nielsen et al. 2023). This can partly arise from narratives framing local communities as the principal drivers of environmental degradation and biodiversity decline, omitting the importance of structural and global social-economic drivers in management strategies (McKinley et al. 2020).

Blue NbS intend to shift this narrative, treating people and nature as partners (IUCN 2020), being designed with, and for, affected communities (O’Leary et al. 2023) (Recommendations 1–3 and 5) to achieve social-ecological objectives (Recommendations 3, 4, and 7). Social adaptive capacity assessments, with input from local communities, can help identify groups particularly vulnerable to climate change and/or ecosystem degradation and suggest ways to help them anticipate, respond, and adapt to these changes (Espinoza Cordova et al. 2024). This information can guide locally appropriate blue NbS design, and the assessment process itself can help embed stakeholders in intervention design, data collection, and social learning (Whitney et al. 2017).

Social adaptive capacity assessments can provide baseline data for monitoring after environmental or management changes. However, case studies applying these assessments for intervention design or monitoring are limited (Whitney et al. 2017). This suggests their potential is underused, highlighting the ongoing need for better integration of social sciences into environmental management (McKinley et al. 2020; O’Leary et al. 2023, 2024). Conducting social adaptive capacity assessments ensures key aspects are addressed in intervention design, including problem conceptualisation, stakeholder roles, and participatory approaches. Designing and implementing these assessments must account for the dynamics between adaptive capacity and social-ecological systems, local experiences, and cross-level interactions to identify key data sources and indicators to inform necessary action (Espinoza Cordova et al. 2024). Although blue NbS aim to prevent further environmental degradation and support ecosystems to persist under climate change, significant uncertainty remains regarding the local impacts of climate change, the best actions to take, and the distribution of costs and benefits across social groups. For people to truly adapt to climate change and environmental degradation, a combination of several strategies is needed, employed over timeframes required and desired by stakeholders (Whitney et al. 2017).

### Recommendation 7: Ensure blue NbS support the reduction of local stressors driving environmental degradation

Reducing local stressors can maintain or improve ecosystems’ ecological conditions and boost resilience (O’Leary et al. 2017; Gove et al. 2023; Trégarot et al. 2024). Following disturbance, healthy ecosystems recover their structure and function more effectively than degraded or fragmented ones (e.g. Faith 2021; Gove et al. 2023). For instance, in Australia’s Palm and Keppel Islands, marine protected areas which removed extractive activities had lower coral disease presence and prevalence following acute and chronic disturbances (e.g. Lamb et al. 2016). Ecologically connected ecosystems are also more resilient since movement corridors facilitate species dispersal for recolonisation (e.g. de Juan et al. 2014) and genetic exchange (e.g. Walsworth et al. 2019). Diverse ecosystems also have higher probability of varied responses to pressure(s) and environmental change, increasing adaptive capacity (e.g. Faith 2021).

To maximise the ability of blue NbS to manage human impacts on marine and coastal ecosystems, intervention design must address both direct (e.g. habitat damage) and/or indirect (e.g. nutrient enrichment) local stressors (Trégarot et al. 2024). This requires an integrated systems approach that considers trade-offs and synergies of co-(dis)benefits (Recommendation 5) and embeds stakeholders within decision-making (Recommendations 1–3 and 6) (Gómez Martín et al. 2020).

Without containing local stressors, blue NbS will not achieve their goals. Because ecosystem resilience relies on healthy ecosystems, management of local stressors is one of the most effective actions we can take for favourable outcomes in uncertain futures (O’Leary et al. 2017; Gove et al. 2023). For example, no-take and multiple-use marine protected areas with adequate enforcement staffing have been shown to increase fish biomass with 97% certainty globally (Gill et al. 2024). With their broader perspective and greater stakeholder engagement, this suggests that blue NbS have the potential to offer advantages over existing ecosystem-based management, provided enabling institutional and enforcement conditions are in place. Suitable locations for blue NbS can be identified through strategic prioritisation of areas needing reactive management to reduce local stressors or likely to benefit from proactive efforts (e.g. Queirós et al. 2021), as well as through local action and opportunity (e.g. Stewart et al. 2020). Once a suitable blue NbS location has been identified, effective regulations focussed on mitigating local stressors need to be implemented, necessitating a clear understanding of local stressors and the baseline condition of the social-ecological system. Information can be gained from sources that include stakeholders, public databases of activities and existing regulations, marine spatial plans, and biodiversity monitoring. To inform future blue NbS strategies, this information should be combined with lessons learned from past management actions, including impacts, successes or failures, and the role of stakeholder involvement in shaping these.

### Recommendation 8: Employ non-invasive methods to collect blue NbS biodiversity data

Measuring biodiversity provides a useful proxy for ecosystem condition, as it reflects ecological integrity, resilience, and responses to environmental change. Defining appropriate indicators that capture different aspects of biodiversity is essential to accurately assess ecosystem health and support the blue NbS evidence base (Recommendation 4). However, data collection methods vary in effectiveness depending on the context and what species need consideration. While past biodiversity assessment has commonly used extractive sampling techniques (e.g. trawls, dredges, grabs), alternative non-invasive methods exist to rapidly collect data at local to seascape scales (Table 1). Examples include use of visual census, imagery (e.g. photography, photogrammetry, videography, remote sensing), environmental DNA (eDNA), and bioacoustics (McGeady et al. 2023; Cabrito et al. 2024; Casal et al. 2024).

**Table 1:** Main advantages and constraints of non-invasive marine and coastal biodiversity data collection methods applied during the MaCoBioS project, based on expert opinion. Note that the list is non-exhaustive, and applicability may vary by ecosystem

Method	Potential uses for ecological monitoring	Main advantages	Main constraints
Visual census (underwater and terrestrial)	Accurate estimation of ecosystem health in relation to species richness and abundance, plus species and habitat distribution Assess ecological change arising from management interventions Identify dominant functional groups	Provides detailed habitat characterisation High level of accuracy Quick and immediate assessment Suitable for marine and terrestrial communities Widespread and historical use allows for considerable comparisons Raw data can be processed into multiple well-known indicators, many of which are easily understood by non-scientists	Ad hoc observations/observer bias limit detailed comparisons of historical and current data Cryptic, pelagic, elusive, or rare species generally not observed Limited accessibility for some ecosystems (hard to reach, including marine ecosystems below ~ 20 m depth) Small-scale coverage May require highly qualified personnel
Underwater drones	Assess ecological condition based on habitat characterisation Assess impact of human activities Monitor epibenthic (visual) species diversity and their behaviour	Can explore deep-sea environments Provides real-time data Versatile as it can be equipped with a variety of sensors Enables large-scale monitoring of continuous data	Tether length and flexibility Generally requires surface support High cost of equipment and needs a skilled technician to operate Time required for video analysis May provide species richness or organism abundance data that are not sufficiently reliable
Environmental DNA (eDNA)	Generates a comprehensive list of species present in an area or look for specific taxa	Can detect poorly monitored species such as rare and cryptic species, or phyla, including bacterial communities Does not require in situ taxonomic specialists for data collection Can provide data over a large spatial scale Can be used in sites not directly accessible to people (e.g. no access, dangerous)	Relies on existing reference databases that can be unbalanced in terms of species representation and/or are difficult to access Mostly generates presence/non-detection data and can deliver false negatives and positives Requires data validation by experts
Bioacoustics	Provides biophonic (sounds of living systems) seascape indicators representing species diversity and abundance within several hundred metres or more Can provide long-term and/or continuous data series Provides anthrophony indicators characterising noise pollution Can reveal certain species behaviour (e.g. activity peaks, reproduction, territoriality)	Accounts for cryptic species in derived diversity indices Can provide data over a large spatial scale Can be used in sites not directly accessible to people (e.g. deep, dangerous) Possibility of long-term records more in line with many ecological processes Can detect behavioural activity	Methodology still under development Sound propagation is not a linear variable Lack of reference data
Aerial drones	Assess ecological change arising from management interventions Assess impact of human activities Can provide seascape scale data Provides spatial and temporal data series	Provides high spatial resolution images Fast to use and cost-effective Highly accurate for habitat monitoring Can be used in sites not directly accessible to people (e.g. no access, dangerous)	Highly dependent on meteorological conditions Requires qualified personnel Specific authorisations may be required Limited spatial and temporal coverage Vulnerable to signal loss
Satellites	Provides data for modelling and mapping to inform management Provides spatial and temporal data series	Generates continuous spatio-temporal data Provides coverage for large areas Long-term data series are available Cost-effective Can be used in sites not directly accessible to people (e.g. no access, dangerous)	Vulnerable to cloud cover when optical sensors are used Coarse spatial resolution for some applications Subtidal coverage can be affected by water quality Not suitable for deep habitats

As with all data collection methods, non-invasive techniques have limitations (Table 1). For example, visual census accuracy depends on observer expertise and naturalist knowledge, which are difficult to standardise within or across studies; video imagery can be poor quality and time-consuming to analyse without machine learning support; eDNA metabarcoding cannot determine species size and abundance; and bioacoustics struggles with most species-level detection due to limited sound libraries (Cabrito et al. 2024). The suitability of an approach varies by deployment area and the monitoring objective. For instance, video imagery effectively characterises biophysical habitats (Haag et al. 2008); eDNA surveys can more accurately detect fish species than extractive methods (He et al. 2023; Cabrito et al. 2024); and bioacoustics shows promise for monitoring fish species and behaviour (Mooney et al. 2020; Cabrito et al. 2024). To improve assessment accuracy and gain comprehensive insights into ecosystem condition, long-term monitoring and evaluation strategies should employ diverse data collection methods to overcome the limitations of individual techniques (Cabrito et al. 2024). As part of this, method selection must consider relevant indicators, deployment ease, required spatial and temporal scales, and availability of expertise, budget, and resources for data collection and processing.

## Conclusion

Links between marine and coastal ecosystems and human health and well-being are increasingly evidenced and recognised. However, biodiversity loss is escalating globally, necessitating transformative social, economic, and technological changes to tackle its underlying drivers. Given the context-dependent nature of blue NbS, there is no one-size-fits all approach and benefits from interventions in one area may not apply elsewhere. Therefore, designing blue NbS requires conscious thought and inclusive engagement to help create socially acceptable interventions that maximise social and ecological benefits and minimise risks, while recognising context-specific constraints, trade-offs, and the possibility of uneven or partial outcomes. Together, these eight recommendations describe how blue NbS can be designed to support strategic, inclusive, and equitable place-based partnerships between nature and people.

## Footnotes

1 www.macobios.eu.
